# Utilization and impact of cardiovascular magnetic resonance on patient management in heart failure: insights from the SCMR Registry

**DOI:** 10.1186/s12968-022-00890-0

**Published:** 2022-11-21

**Authors:** Idan Roifman, Michael Hammer, John Sparkes, Erica Dall’Armellina, Raymond Y. Kwong, Graham Wright

**Affiliations:** 1 Schulich Heart Program, Sunnybrook Health Sciences Center, University of Toronto, Toronto, On Canada; 2 University of Leeds, England, UK; 3 Cardiovascular Division, Department of Medicine, Brigham and Women’s Hospital, Harvard Medical School, Boston, USA; 4 Echocardiography laboratory, Sunnybrook Health Sciences Center Scientist, Sunnybrook Research Institute, Institute for Clinical Evaluative Sciences, Department of Medicine, Canadian Society for Cardiovascular Magnetic Resonance Director, University of Toronto, Toronto, Canada

**Keywords:** Heart failure, Registry, Real World, Impact of CMR

## Abstract

**Background:**

Cardiovascular magnetic resonance (CMR) is an important diagnostic test used in the evaluation of patients with heart failure (HF). However, the demographics and clinical characteristics of those undergoing CMR for evaluation of HF are unknown. Further, the impact of CMR on subsequent HF patient care is unclear. The goal of this study was to describe the characteristics of patients undergoing CMR for HF and to determine the extent to which CMR leads to changes in downstream patient management by comparing pre-CMR indications and post-CMR diagnoses.

**Methods:**

We utilized the Society for Cardiovascular Magnetic Resonance (SCMR) Registry as our data source and abstracted data for patients undergoing CMR scanning for HF indications from 2013 to 2019. Descriptive statistics (percentages, proportions) were performed on key CMR and clinical variables of the patient population. The Fisher’s exact test was used when comparing categorical variables. The Wilcoxon rank sum test was used to compare continuous variables.

**Results:**

3,837 patients were included in our study. 94% of the CMRs were performed in the United States with China, South Korea and India also contributing cases. Median age of HF patients was 59.3 years (IQR, 47.1, 68.3 years) with 67% of the scans occurring on women. Almost 2/3 of the patients were scanned on 3T CMR scanners. Overall, 49% of patients who underwent CMR scanning for HF had a change between the pre-test indication and post CMR diagnosis. 53% of patients undergoing scanning on 3T had a change between the pre-test indication and post CMR diagnosis when compared to 44% of patients who were scanned on 1.5T (p < 0.01).

**Conclusion:**

Our results suggest a potential impact of CMR scanning on downstream diagnosis of patients referred for CMR for HF, with a larger potential impact on those scanned on 3T CMR scanners.

**Supplementary Information:**

The online version contains supplementary material available at 10.1186/s12968-022-00890-0.

## Background

Heart failure (HF) is a global public health problem affecting at least 26 million people worldwide and is increasing in prevalence [1–3]. Cardiovascular magnetic resonance (CMR) imaging is an important diagnostic test in the assessment of patients with HF [4–18]. However, the demographics and clinical characteristics of those undergoing CMR for the evaluation of HF are unknown. Further, the impact of CMR on subsequent HF patient care is unclear. Much of the current available evidence in this area is built upon small single center studies with widely varying results and conclusions. For example, CMR has been reported to lead to a change in patient management in anywhere between 16 and 65% of studies [19–21]. One way in which CMR can lead to a change in patient management is by providing valuable diagnostic information that leads to a change in the understanding of the etiology of HF post-test, when compared to pre-test [19] [20, 21]. This diagnostic information can help inform downstream treatment decisions and impact outcomes. However, the extent to which CMR can impact HF management in this manner is currently unclear. A systematic, multi-center, evaluation of the impact of CMR on patient management is required to shed further light onto these issues.

The objectives of this paper are to utilize the Society for Cardiovascular Magnetic Resonance (SCMR) Registry in order to:

1. Describe patient demographics, clinical and CMR scanning characteristics of patients undergoing CMR for HF; and.
2. Determine the extent to which CMR is associated with changes in downstream patient management by comparing the pre-CMR indication and the post-CMR diagnostic information.

## Methods

### Data source

The data source was the SCMR Registry. This Registry was created by the SCMR in 2013 and the SCMR has continued to support this registry since its creation. For this project, data were abstracted from Jan 1, 2013- Dec 31, 2019. The overarching vision of the SCMR was to provide a central platform to demonstrate the impact of CMR on patient outcomes and clinical care. One of its stated goals is the determination of the downstream impact of CMR on diagnostic clinical decision making [22]. At the time that this project was performed, the SCMR Registry contained demographic and CMR data from 13 centers across the world. Site participation was invited and advertised by the SCMR at multiple forums, including at the SCMR Annual Scientific Sessions. Data for the registry were collected by individual sites retrospectively on consecutive patients who underwent clinically indicated CMR scans and then entered into the SCMR registry by those sites.

## Identification of the patient population and cohort creation

CMRs were identified in the SCMR Registry if they were performed for a HF related pre-test indication. Specifically, patients with left ventricular ejection fraction (LVEF) < 55% with the following pre-CMR suspected indications were included: amyloidosis, coronary artery disease (CAD) AND categorized as having LV dysfunction, arrhythmogenic right ventricular dysplasia (ARVC)/cardiomyopathy, cardiomyopathy, arrhythmic disease, Friedrich’s ataxia, hypertrophic cardiomyopathy (HCM) or hemochromatosis. Derivation of the above-mentioned algorithm for patient selection involved the following steps: First, a draft list of indications was selected a priori by the lead author (IR) and a co-author who was largely responsible for establishing the SCMR Registry and who was intimately familiar with its structure and data (RK). This list was based on the principle of balancing the detection of the largest number of patients who received a CMR for the indication of HF whilst minimizing inclusion of patients who were not scanned for HF related indications. It is for this reason that we included only those patients with reduced LVEF (LVEF < 55%) in the cohort. While this list excluded those with HF with preserved ejection fraction, we believed that removing the LVEF inclusion condition would contaminate our data by including many patients who were not scanned for HF indications. Next, as this an SCMR initiated paper, the proposal including the criteria for patient selection was reviewed and approved by the SCMR’s Science Committee. Following this initial approval, the dataset/cohort for the project was created from the larger SCMR Registry based on the approved patient selection criteria. Subsequently, the data and various iterations of the paper were approved by the SCMR’s Publications Committee. The final paper, prior to submission to this journal was approved by the SCMR’s Board of Trustees.

## Determination of whether CMR was associated with changes in downstream management

We determined that a change in management was associated with the CMR if there was a change between the initial indication for the CMR and the subsequent diagnosis after the CMR was completed. The ‘indication’ field was an existing variable in the SCMR Registry. We then reviewed, for each subject, the text field where the final conclusions from the CMR scan were inserted. Using our clinical judgement, we determined if there was a clinically relevant change from the original pre-CMR indication, in a method similar to that used in other studies [21]. The initial screen was done by a senior cardiovascular disease resident (MH). In terms of training, this resident had completed a cardiology residency program (i.e. was a board certified cardiologist in Canada) and was undergoing a fellowship in advanced cardiac imaging at the time of the data review. As such, the resident was familiar with the role of CMR in the management of cardiovascular disease. All results were subsequently reviewed by a level 3 trained SCMR cardiologist and Fellow of the SCMR (IR).

### Statistical analysis

Descriptive statistics (percentages, proportions) were performed on key CMR and clinical variables of the patient population. The Fisher’s exact test was used when comparing categorical variables. Specifically, this test was used when comparing the change rate between patients undergoing CMR at 1.5 vs. 3T, between males and females, between patients of different body mass index (BMI) categories, and between patients with and without late gadolinium enhancement (LGE). The Wilcoxon rank sum test was used to compare continuous variables. P values of < 0.05 were considered statistically significant.

## Results

There were 6,654 patients in the registry who underwent CMR for the indication of HF during our study period. However, 2,817 patients were excluded because they did not have data entered regarding their pre-CMR indication and/or post CMR diagnosis to allow us to evaluate whether or not receipt of CMR was associated with a change in management. Thus, 3,837 patients ultimately remained in our cohort. Of these, 94% of the CMRs were performed in the United States with 68% occurring at one site (see Table 1). Other countries with significant contributions to the SCMR Registry include China (n = 182, 4.7% and South Korea (n = 41, 1.1%). India contributed 3 cases.

**Table 1:** Distribution of sites contributing to the Society for Cardiovascular Magnetic Resonance (SCMR) Registry

Country	Site Number	No. of Patients	Percentage
United States	87	1	< 0.1%
United States	70	1	< 0.1%
India	54	3	0.1%
United States	25	7	0.2%
United States	63	31	0.8%
South Korea	68	41	1.1%
United States	86	56	1.5%
United States	36	116	3.0%
United States	85	145	3.8%
China	18	182	4.7%
United States	2	262	6.8%
United States	8	376	9.8%
United States	1	2,616	68.2%
	Total:	3,837	

## Baseline patient characteristics

Baseline patient characteristics are summarized in Table 2. Median age of subjects was 59.3 years (IQR, 47.1, 68.3 years) the median BMI was 27. 1 kg/m^2^ (IQR, 23.8, 31.5 kg/m2). Women constituted 67% of the patients. In terms of major cardiovascular risk factors, 49% of patients had hypertension, 18% had diabetes, 21% were active smokers and 11% had a family history of premature CAD. There were 36% of patients who had evidence of overt CAD (prior myocardial infarction, percutaneous coronary interventions and/or coronary artery disease). With regards to cardiac function, the median LVEF was 41% (IQR 29%, 50%) and the median right ventricular ejection fraction (RVEF) was 48% (IQR 39%, 54%). 3,540 patients had data entered regarding LGE positivity (positive or negative). Overall, 54% of patients were LGE positive. Patterns of LGE (for example ischemic vs. non-ischemic) as well as LGE quantity were not available in the data.

**Table 2:** Characteristics of the patient population

	*Mean ± SD*	*Median*	*Q1*	*Q3*	*% missing values*
Age, years	57.0 ± 16.1	59.3	47.1	68.3	0.3%
Height, meters	1.72 ± 0.11	1.73	1.65	1.80	1.1%
Weight, kilograms	83.8 ± 21.9	81.5	68.0	96.5	0.4%
BMI (kilograms/m^2^)	28.1 ± 6.5	27.1	23.8	31.5	1.1%
	Percentage				% *missing values*
Female sex	67%				0%
**Cardiac function**					
		*Median*	*Q1*	*Q3*	*% missing values*
LVEDV, mL		195	156	250	15%
LVESV, mL		112	83	169	16%
LVEF, %		41	29	50	16%
LVM, g		128	100	161	25%
RVEDV, mL		147	116	185	22%
RVESV, mL		77	57	106	22%
LVEDVI, mL/m^2^		100	82	127	16%
LVESVI, mL/m^2^		57	43	86	16%
LVSV, mL		74	58	91	16%
LVEDD, mm		60	54	67	13%
LVESD, mm		47	40	57	14%
LVMI, g/m2		65	53	81	25%
RVEDVI, mL/m2		76	61	92	23%
RVESVI, mL/m2		39	30	54	23%
RVSV, mL		67	51	84	22%
RVEF, %		48	39	54	22%
**Cardiovascular history**					
	Percentage				*% missing values*
History of myocardial infarction	18%				11%
History of percutaneous coronary intervention	12%				11%
History of coronary artery bypass grafting	6%				10%
History of hypertension	49%				9%
History of diabetes	18%				10%
History of heart failure	37%				11%
History of dyslipidemia	39%				10%
History of smoking	21%				8%
History of peripheral vascular disease	4%				12%
Family history of coronary artery disease	11%				14%

*BMI*: Body Mass Index *LVEDV*: Left ventricular (LV) end-diastolic volume, *LVEDVI*: LV end-diastolic volume indexed to body surface area, *LVESV*: LV end-systolic volume, *LVESVI*: LV end-systolic volume indexed to body surface area, *LVSV*: LV stroke volume, *LVEF*: LV ejection fraction, *LVEDD*: LV end-diastolic dimension, *LVESD*: LV end-systolic dimension, *LVM*: LV mass, *LVMI*: LV mass indexed to body surface area, *RVEDV*: Right ventricular (RV) end-diastolic volume, *RVESV*: RV end-systolic volume; *RVSV*: RV stroke volume, *RVE*F: RVejection fraction, *RVEDVI*: RV end-diastolic volume indexed to body surface area, *RVESVI*: RV end-systolic volume indexed to body surface area

## Sequences utilized in the CMRs

Table 3 summarizes the pulse sequences employed in patients referred for CMR for HF. 94% of patients underwent cine balanced steady state free precession (bSSFP) imaging whilst 90% underwent LGE imaging, 43% underwent T2 weighted imaging and 22% underwent T1 mapping sequences.

**Table 3:** CMR Pulse Sequences

Variable Name	1.5T (N = 1,217)	3T (N = 2,385)	All Scans (N = 3,837)
Balanced steady state free precession	89.6%	100.0%	94.2%
T2 weighted imaging	37.6%	50.6%	42.5%
T1 mapping	4.5%	33.5%	21.6%
T2 mapping	2.8%	23.6%	14.7%
T2 *	20.2%	33.1%	26.6%
Stress perfusion	22.4%	16.3%	17.6%
Late gadolinium enhancement	86.9%	96.7%	90.4%

## Field strength and image quality

There were 2,385 patients who were scanned on a 3T CMR system (62.1%) vs. 1,217 patients who were scanned at 1.5 T(31.7%). Regarding CMR image quality; 88% of scans were ranked as ‘good’ or ‘excellent’ with 12% ranked as ‘poor’ or ‘fair’. These clinical judgements regarding image quality were site-based assessments and were performed qualitatively by the reading physician. There were no preset criteria to distinguish what constituted ‘excellent’, ‘good’, ‘fair’ or ‘poor’ image quality.

## Initial indications and diagnosis following CMR scanning

The top 6 indications for CMRs performed in the SCMR Registry were: Cardiomyopathy, etiology not yet diagnosed (NYD) (1,776; 46.2%), CAD/ischemia/viability (1,230, 32.1%), ARVC (423, 11.0%), HCM (146, 3.8%), arrhythmic substrate (136, 3.5%) and amyloidosis (97, 2.5%). Overall, in 1,892 (49%) of patients, there was a change between the initial pre-test indication and the post CMR diagnosis. When broken down by indication, CMR was associated with changes between indication and post-CMR diagnosis in 333 (79%) of those referred for ARVC, 1,114 (63%) of those referred for cardiomyopathy, 136 (49%) of those referred for arrhythmic disease, 66 (45%) of those undergoing CMR for HCM, 25 (26%) undergoing scanning for amyloidosis and 270 (22%) of those undergoing CMR for CAD. Table 4 summarizes the nature of these changes amongst those who had CMRs performed for the top 6 indications. Table 5 stratifies the change rate between the pre-CMR indication and post-CMR diagnosis according to site. The largest contributing American site had a similar change rate (51%) to that of the entire patient population. The second largest contributing site, also from the United States, similarly had a change rate of 50%. In total, there were 3,611 patients who received CMRs at American sites and they had an overall change rate of 50%. The country that contributed the second largest number of patients was China. They contributed 182 patients and reported a change rate of 52%, similar to the overall and American rate. The country that reported the lowest change rate was South Korea (15%).

**Table 4:** Pre-CMR indications and post CMR diagnoses

Pre-CMR indication	Number of patients with pre-CMR diagnoses (%)	Most Common Post-CMR diagnoses	Number of patients with post-CMR diagnoses (%)
**Amyloidosis of the heart**			
Total	97		
No change in diagnosis	72 (74%)	Same as pre-CMR diagnosis	72 (100%)
Change in diagnosis	25 (26%)		
		Non-ischemic cardiomyopathy	13 (52%)
		Coronary artery disease	4 (16%)
		Left ventricular hypertrophy	3 (12%)
		No amyloidosis	4 (16%)
		Possible hypertrophic cardiomyopathy	1 (4%)
**Arrhythmic Disease**			
Total	136		
No change in diagnosis	70 (51%)	Same as pre-CMR diagnosis	70 (100%)
Change in diagnosis	66 (49%)		
		Non-ischemic cardiomyopathy	44 (67%)
		Coronary artery disease	9 (14%)
		Possible myocarditis	4 (6%)
		Myocarditis	1 (2%)
		Hypertrophic cardiomyopathy	1 (2%)
		Left ventricular non-compaction	1 (2%)
		Coronary artery disease and non-ischemic cardiomyopathy	1 (2%)
		Cardiomyopathy and ventricular septal defect	1 (2%)
		Possible transplant rejection	1 (2%)
		No arrhythmic substrate	3 (5%)
**ARVC**			
Total	423		
No change in diagnosis	90 (21%)	Same as pre-CMR diagnosis	90 (100%)
Change in diagnosis	333 (79%)		
		Non-ischemic cardiomyopathy	131 (39%)
		No ARVC	60 (18%)
		Borderline left ventricular function without other significant abnormalities	96 (29%)
		Coronary artery disease	13 (4%)
		Myocarditis	3 (1%)
		ARVC and cardiomyopathy	3 (< 1%)
		LV non-compaction	5 (2%)
		Infiltrative cardiomyopathy	2 (< 1%)
		Likely inflammatory cardiomyopathy	1 (< 1%)
		Left ventricular hypertrophy	2 (< 1%)
		Ventricular septal defect	1 (< 1%)
		Right atrial dilatation without ARVC	5 (2%)
		Other diagnoses	11 (3%)
**Cardiomyopathy NYD**			
Total	1,776		
No change in diagnosis	662 (37%)	Same as pre-CMR diagnosis	662 (100%)
Change in diagnosis	1,114 (63%)		
		Coronary artery disease	240 (22%)
		Non-ischemic cardiomyopathy	484 (43%)
		Myocarditis	52 (5%)
		Left ventricular non-compaction	42 (4%)
		Borderline LV function without other significant abnormalities	70 (6%)
		Sarcoidosis	34 (3%)
		ARVC	9 (< 1%)
		Hypertrophic cardiomyopathy	9 (< 1%)
		Coronary artery disease and non-ischemic cardiomyopathy	16 (1%)
		Infiltrative cardiomyopathy	26 (2%)
		Hemochromatosis	2 (< 1%)
		Other diagnoses	130 (12%)
**Coronary Artery Disease**			
Total	1,230		
No change in diagnosis	960 (78%)	Same as pre-CMR diagnosis	960 (100%)
Change in diagnosis	270 (22%)		
		Non-ischemic cardiomyopathy	154 (57%)
		Borderline left ventricular function without other significant abnormalities	42 (16%)
		No coronary artery disease	9 (3%)
		Myocarditis	12 (4%)
		Coronary artery disease and non-ischemic cardiomyopathy	11 (4%)
		Sarcoidosis	4 (1%)
		Left ventricular non-compaction	4 (1%)
		Infiltrative cardiomyopathy	8 (3%)
		Hypertrophic cardiomyopathy	2 (< 1%)
		Hemochromatosis	1 (< 1%)
		Other diagnoses	23 (9%)
**Hypertrophic Cardiomyopathy**			
Total	146		
No change in diagnosis	80 (55%)	Same as pre-CMR diagnosis	80 (100%)
Change in diagnosis	66 (45%)		
		Non-ischemic cardiomyopathy	22 (33%)
		Coronary artery disease	11 (17%)
		Borderline left ventricular function without other significant abnormalities	12 (18%)
		Infiltration/amyloid	6 (9%)
		Left ventricular hypertrophy	3 (5%)
		No hypertrophic cardiomyopathy	5 (8%)
		Left ventricular non-compaction	6 (9%)
		Concomitant hypertrophic cardiomyopathy and coronary artery disease	1 (2%)

**Legend. ARVC**: Arrhythmogenic right ventricular cardiomyopathy, **NYD**: Not yet diagnosed

**Table 5:** Stratification by site according to the rate of change between pre-CMR indication and post-CMR diagnosis

Country of the site performing the CMR	Site Number	No. of Patients	No change between pre-CMR indication and post-CMR diagnosis	Change between pre-CMR indication and post-CMR diagnosis	Percentage with change between pre-CMR indication and post-CMR diagnosis
United States	1	2,616	1,286	1,330	51%
United States	8	376	187	189	50%
United States	2	262	145	117	45%
United States	85	145	67	78	54%
United States	36	116	69	47	41%
United States	86	56	43	13	23%
United States	63	31	17	14	45%
United States	25	7	5	2	29%
United States	70	1	1	0	0%
United States	87	1	1	0	0%
China	18	182	88	94	52%
South Korea	68	41	35	6	15%
India	54	3	1	2	67%
Total		3,837	1,945	1,892	49%

Patients scanned at 3T had higher change rates vs. those scanned at 1.5 T (53% vs. 44% respectively, p < 0.001). Those scanned on a 3T CMR system had their images rated at ‘excellent’ or ‘good’ 87% of the time versus 90% of scans performed at 1.5 T (p < 0.001). Males had a higher rate of change following CMR (52% vs. 48% respectively, p = 0.02). There was a non-significant trend towards a higher rate of change amongst those of normal weight/underweight vs. those who were overweight. Those with BMI < 25 kg/m^2^ had a change rate 51% of the time when compared with a change rate of 49% in those with a BMI > = 25 kg/m^2^ (p = 0.23). Amongst patients undergoing 3T CMR scanning, there was no significant difference regarding change rate between those with BMIs < 25 kg/m^2^ (55%), 25-29.9 kg/m^2^ (53%) and > 30 kg/m^2^ (48%), p = 0.54. Among patients who were LGE positive, CMR was associated with a change between initial indication and post-CMR diagnosis in 44% of the cases vs. 56% of the cases for those who were LGE negative (p < 0.001).

## Sensitivity analysis

To minimize the risk of bias caused by data submitted by small contributing sites, we repeated our main analysis after removing sites that contributed < 10 patients. After removal of these patients, 3,825 patients remained from 9 sites. There was no significant difference in our results with 1,888 patients having a change between the initial indication and post-CMR diagnosis (49%) and 1,937 patients (51%) not having such a change.

## Comparison of cohort patients with excluded patients

Supplemental Table 1 compares patients ultimately included in the cohort with those excluded. Of note, there was no significant difference in terms of median age, height, weight or BMI between the two groups (p-value > 0.05). Patients included in the cohort were slightly more likely to be female (67% vs. 64%, p-value = 0.01). There were also no significant differences in CMR characteristics between those patients who were included and those excluded. However, patients included in the study had significantly higher rates of major cardiovascular risk factors when compared to those who were excluded. The excluded patients also had significantly higher percentages of missing values for these clinical parameters.

## Discussion

Our analysis of 3,837 consecutive patients from the SCMR Registry referred for CMR for the evaluation of HF reveals a median age of approximately 59 years. The majority of CMR scans were performed on women. Almost 2/3 of the patients were scanned on 3T magnets. CMR was associated with changes between the initial indication and the post-CMR diagnosis in nearly one half of patients. Patients undergoing scanning on 3T were significantly more likely to have a subsequent change when compared to those scanned on 1.5T, despite 1.5T scans having slightly better rated image quality.

Despite being considered the gold standard diagnostic test for patients with HF, there is little available evidence that receipt of CMR leads to changes in downstream patient management. In a single center study, White et al. examined 82 patients undergoing CMR for the diagnosis of arrhythmic substrate [21]. They reported that 50% of patients had a change in management, as identified by a change in diagnosis after CMR scanning. In a larger single center study, Abbasi et al. studied 150 subjects with LVEF < 50%. They reported a downstream change in management of 52% after the CMR was performed [19]. Our large multi-center study of > 3,000 patients from the SCMR Registry reported a similar rate of change in management after CMR scanning compared to the two aforementioned studies. These results are clinically important as they confirm the impact of CMR in the management of a large number of HF patients across multiple centers and countries. Further, our results reporting that CMR impact on management was more likely after scanning on a 3T magnet are interesting. Although our study was not designed to evaluate the reason underlying this finding, we speculate that it may be related to improved diagnostic quality of some tissue characterization sequences such as LGE, T2 weighted imaging and T1 mapping, despite the fact that our data does not report overall higher image quality for 3T CMR. Since our registry recorded data for overall CMR scan image quality but not for individual sequences, we are unable to test this hypothesis. It is also important to note that the main contributing US site exclusively uses 3T CMR systems. This may have been an important contributor to the differential impact that we observed for patients scanned on 1.5 vs. 3T in terms of diagnostic change.

Most of the patient characteristics that we reported in our cohort were similar to previously published work from other HF registries. For example, the Swedish Heart Failure Registry reported a hypertension prevalence of 52% (vs. 49% in our cohort) and a diabetes prevalence of approximately 20% (vs. 18% in our cohort) [23]. One important difference is that the mean age in the Swedish registry was approximately 77 years versus our mean of approximately 57 years. One potential explanation for this discrepancy is that all the patients in our cohort, by definition, must have received a CMR. This is in comparison to the Swedish registry where only a small proportion of the patients underwent CMR scanning. There is the potential for selection bias in our study as physicians may be more likely to order CMRs on younger, healthier patients who may tolerate the CMR diagnostic test better and in whom there may be more therapeutic options. Another important finding of ours that differs from prior research is that approximately 2/3 of the patients in our cohort were female. Prior research has reported that prevalence rate of HF is similar in men and women, raising the question of why our cohort contained such a high proportion of women [24–26]. A possible contributor to this is that women with HF have been shown to ascribe more positive meaning to their illness [24, 27–29], perhaps making them more likely to agree to undergo more extensive diagnostic evaluation, including with a CMR. Indeed, there are limited data to suggest that women are more likely to receive advanced imaging tests for the evaluation of possible CAD [30]. If this was the case for the SCMR Registry, it is possible that this attribute led to a selection bias that ultimately culminated in a significantly higher percentage of women being included.

Although most sites had similar rates of changes between the initial indication and the post-CMR diagnosis, some sites had significantly lower rates. The lowest rate of change occurred in the South Korean contributing site. Given the overall small numbers of patients scanned at this site (n = 41), it is difficult to draw firm conclusions about possible reasons for this low rate. The pre-CMR indications were different at this site when compared to the overall cohort with only approximately 56% being performed for cardiomyopathy NYD or CAD/ischemia (vs. approximately 78% of the total CMRs) and 22% performed for arrhythmic substrate (vs. approximately 4% of the total). Further, unlike the overall cohort, the South Korean site scanned patients predominantly at 1.5T (71%), which was associated with lower rates of diagnostic change post CMR in our study.

In a comparison of patients who were ultimately included in our cohort with those who were excluded, the former had a significantly higher prevalence of most major cardiovascular risk factors when compared to the latter despite no significant differences in terms of age, weight, BMI or cardiac function parameters. However, it is important to note that those patients excluded from our study also had a significantly higher percentage of missing values for cardiovascular history/risk factor parameters. Indeed, the reason those patients were excluded was because they had insufficient data for us to make a determination of whether or not there was a change between the initial pre-CMR indication and the post-CMR diagnosis.

## Limitations

This study must be interpreted in the context of its limitations. First, there was limited data granularity in the registry. For example, we did not have data on treatment and downstream clinical outcomes. We recognize that a change between the pre-CMR indication and post CMR diagnosis is only one aspect in the assessment of a change in management and we lack direct data on downstream treatment decisions and outcomes. Furthermore, CMR does have value in confirming diagnoses and therefore evaluating for a change between the pre-test indication and the post-test diagnosis is an imperfect surrogate in the overall assessment of whether a change in management occurred. With that said, the enhanced ability to detect new or alternate myocardial disease processes is valuable and uniquely differentiates CMR from many other imaging modalities. This has been recognized by other groups who used similar surrogates in their work evaluating the clinical impact of CMR [21]. In another example of our lack of data granularity, we did not have access to the raw images and relied on data input from CMR reports from the participating sites. Our work highlights the importance of improving data capture/granularity in the SCMR Registry to help facilitate future research. Second, there were significant missing data in a number of parameters (summarized in Tables 2 and 3). Future SCMR Registry related quality improvement processes should focus on increasing site compliance with data entry in order to produce a more complete and accurate registry. Third, although we used the global SCMR Registry, the vast majority of the data were from the United States with most of that data derived from one American site. However, it is important to note that for our study, the one dominant site had a very similar change rate (51%) when compared to the entire cohort (49%). Nonetheless, we recognize that using data primarily from one site may affect generalizability and future efforts should focus on diversifying data input from other sites and countries. Fourth, some parameters were qualitative and were reliant solely on site assessments (such as the evaluation of image quality). Future SCMR Registry efforts should focus on harmonizing or standardizing such qualitative data across sites. Finally, due to the design of our study, it was not possible to blind the resident who evaluated whether or not a change occurred to the initial pre-CMR indication. This may have led to ascertainment bias. In order to minimize the impact of this potential bias, a second reviewer who is a Fellow of the SCMR and who has > 10 years of experience in clinical cardiology, CMR clinical research and the interpretation of large datasets, reviewed and confirmed all the findings after initial review by the resident.

## Conclusion

In our study of 3,837 SCMR Registry patients undergoing CMR for the evaluation of HF, we found that CMR was associated with a change between the pre-test indication and post-CMR diagnosis in 49% of cases, suggesting a potential impact on patient management. The rate of change occurred more commonly for patients scanned at 3T.

## Electronic supplementary material

Below is the link to the electronic supplementary material.

Supplementary Material 1

## Data Availability

Only sites participating in the registry have access to this data.

## Declarations

2. Given the retrospective nature of the current data spanning more than a decade, obtaining informed consent from each patient was not logistically feasible, and a waiver for signing informed consent was obtained from the IRB of each participating site.

IRBs of sites that have contributed data:

Brigham and Women’s Hospital: Partners Human Research Committee.

Central Utah Clinic: Review of this project has been waived by the regulatory bodies of Revere Health, Provo, Utah, as only non-identifiable information was shared.

National Institutes of Health: The IRB of the National Heart, Lung, and Blood Institute, Division of Intramural Research.

Ohio State University: OSU Human Subjects Review Committee.

Oklahoma Heart Institute: Hillcrest Medical Center IRB.

University of Oxford: The UK’s Health Research Authority states that research involving previously collected non-identifiable information does not require research ethics committee review.

St. Francis Hospital: The St. Francis Hospital Institutional Review Board.

University of South Florida: Tampa General Hospital Institutional Review Board.

West China Hospital: Ethics Committee at West China Hospital.

Wilford Hall Medical Center: San Antonio Military Medical Center Institutional Review Board.

## List of abbreviations

**RVC**: Arrhythmogenic right ventricular cardiomyopathy
**BMI**: Body mass index
**bSSFP**: Balanced steady state free precession
**CAD**: Coronary artery disease
**CMR**: Cardiovascular magnetic resonance
**HCM**: Hypertrophic cardiomyopathy
**HF**: Heart failure
**LGE**: Late gadolinium enhancement
**LVEF**: Left ventricular ejection fraction
**NYD**: Not yet diagnosed
**RVEF**: Right ventricular ejection fraction
**SCMR**: Society for Cardiovascular Magnetic Resonance
