# Supplementary material for: Utilization and impact of cardiovascular magnetic resonance on patient management in heart failure: insights from the SCMR Registry
Source: J Cardiovasc Magn Reson. 2022 Nov 21;24:65. doi: 10.1186/s12968-022-00890-0 (PMC9677679; doi:10.1186/s12968-022-00890-0)
Supplement: Supplementary file 1 — Supplementary Material 1 [file 12968_2022_890_MOESM1_ESM.docx]

| **Supplemental Table 1. Comparison of patients included in the cohort with those excluded** | | | |
| --- | --- | --- | --- |
|  | **Patients included in cohort (n= 3,837)** | **Excluded patients (n= 2,817)** | p-value |
| **Patient demographics** |  |  |  |
| Median age, years | 59.3 | 52.3 | 0.98 |
| Median height, m | 1.73 | 1.66 | 0.48 |
| Median weight, kg | 81.5 | 66.0 | 0.41 |
| Median body mass index, kg/m2 | 27.1 | 24.1 | 0.67 |
|  |  |  |  |
| Female sex percentage | 67% | 64% | 0.01 |
|  |  |  |  |
| **Cardiac function** | Median | Median |  |
|  |  |  |  |
| LVEDV, mL | 195 | 192 | 0.92 |
| LVESV, mL | 112 | 112 | 0.95 |
| LVEF, % | 41 | 39 | 0.84 |
| LVM, g | 128 | 130 | >0.99 |
| RVEDV, mL | 147 | 141 | 0.89 |
| RVESV, mL | 77 | 74 | 0.92 |
| LVEDVI, mL/m2 | 100 | 105 | 0.71 |
| LVESVI, mL/m2 | 57 | 62 | 0.26 |
| LVSV, mL | 74 | 70 | 0.95 |
| LVEDD,mm | 60 | 59 | 0.52 |
| LVESD,mm | 47 | 46 | 0.50 |
| LVMI, g/m2 | 65 | 72 | 0.62 |
| RVEDVI, mL/m2 | 76 | 80 | 0.67 |
| RVESVI, mL/m2 | 39 | 42 | 0.39 |
| RVSV, mL | 67 | 62 | 0.94 |
| RVEF, % | 48 | 47 | > 0.99 |
|  |  |  |  |
| **Cardiovascular history and risk factors** |  |  |  |
|  | Percent | Percent |  |
| History of myocardial infarction | 18% | 6% | <0.001 |
| History of percutaneous coronary intervention | 12% | 6% | <0.001 |
| History of coronary artery bypass grafting | 6% | 2% | <0.001 |
| History of hypertension | 49% | 25% | <0.001 |
| History of diabetes | 18% | 9% | <0.001 |
| History of congestive heart failure | 37% | 9% | <0.001 |
| History of dyslipidemia | 39% | 14% | <0.001 |
| History of smoking | 21% | 20% | <0.001 |
|  |  |  |  |
| **Missing values for cardiovascular history and risk factors** (percent) | | |  |
|  | Percent | Percent |  |
| History of myocardial infarction | 11% | 15% | 0.004 |
| History of percutaneous coronary intervention | 11% | 15% | <0.001 |
| History of coronary artery bypass grafting | 10% | 16% | <0.001 |
| History of hypertension | 9% | 13% | <0.001 |
| History of diabetes | 10% | 14% | 0.01 |
| History of congestive heart failure | 11% | 18% | <0.001 |
| History of dyslipidemia | 10% | 14% | <0.001 |
| History of smoking | 8% | 13% | <0.001 |

**Legend.** **LVEDV:** Left ventricular (LV) end-diastolic volume, **LVEDVI:** LV end-diastolic volume indexed to body surface area, **LVESV:** LV end-systolic volume, **LVESVI:** LV end-systolic volume indexed to body surface area, **LVSV:** LV stroke volume, **LVEF:** LV ejection fraction, **LVEDD:** LV end-diastolic dimension, **LVESD:** LVend-systolic dimension, **LVM:** LV **mass, LVMI:** LV mass indexed to body surface area, **RVEDV:** Right ventricular (RV) end-diastolic volume, **RVESV:** RV end-systolic volume; **RVSV:** RV stroke volume, **RVEF:** RV ejection fraction, **RVEDVI:** RV end-diastolic volume indexed to body surface area, **RVESVI:** RV end-systolic volume indexed to body surface area
